# Dissecting the heterogeneity of macrophage activation syndrome

**DOI:** 10.1186/1546-0096-12-S1-P54

**Published:** 2014-09-17

**Authors:** Sergio Davì, Francesca Minoia, AnnaCarin Horne, Francesca Bovis, Erkan Demirkaya, Jonathan Akikusa, Nuray Aktay Ayaz, Patrizia Barone, Bianca Bica, Isabel Bolt, Luciana Breda, Zane Davidsone, Carmen De Cunto, Jaime De Inocencio, Sandra Enciso, Romina Gallizzi, Thomas Griffin, Teresa Hennon, Gerd Horneff, Maka Ioseliani, Michael Jeng, Agneza Marja Kapovic, Bianca Lattanzi, Jeffrey M Lipton, Silvia Magni-Manzoni, Clarissa Nassif, Ingrida Rumba, Claudia Saad Magalhaes, Sulaiman Al-Mayouf, Wafaa Mohammed Sewairi, Kimo C Stine, Olga Vougiouka, Lehn Weaver, Mabruka Ahmed Zletni, Nicola Ruperto, Alberto Martini, Randy Q Cron, Angelo Ravelli

**Affiliations:** 1 Istituto G. Gaslini, Genova, Italy; 2 Investigator Consortium for MAS Classification Criteria (ICMCC), Stockholm, Sweden; 3 ICMCC, Ankara, Turkey; 4 ICMCC, Melbourne, Australia; 5 ICMCC, Istanbul, Turkey; 6 ICMCC, Catania, Italy; 7 ICMCC, Rio de Janeiro, Brazil; 8 ICMCC, Zurich, Switzerland; 9 ICMCC, Chieti, Ital; 10 ICMCC, Riga, Latvia; 11 ICMCC, Buenos Aires, Argentina; 12 ICMCC, Madrid, Spain; 13 ICMCC, Mexico City, Mexico; 14 ICMCC, Messina, Italy; 15 ICMCC, Charlotte, USA; 16 ICMCC, Buffalo, USA; 17 ICMCC, Sankt Augustin , Germany; 18 ICMCC, Tbilisi, Georgia; 19 ICMCC, Stanford, USA; 20 ICMCC, Zagreb, Croatia; 21 ICMCC, Ancona, Italy; 22 ICMCC, New York, USA; 23 ICMCC, Roma, Italy; 24 ICMCC, Belo Horizonte; 25 ICMCC, Botucatu, Brazil; 26 ICMCC, Riyadh, Saudi Arabia; 27 ICMCC, Little Rock, USA; 28 ICMCC, Athens, Greece; 29 ICMCC, Philadelphia, USA; 30 ICMCC, Tripoli, Libya; 31 ICMCC, Birmingham, USA

## Introduction

Macrophage activations syndrome (MAS) in systemic juvenile idiopathic arthritis (sJIA) can pursue a rapidly fatal course. However, diagnosis is often challenging as MAS may be mimicked by confusable conditions, such as flares of sJIA or systemic infections. In addition, the clinical spectrum of MAS is known to be heterogeneous.

## Objectives

To seek insights into the heterogeneity of MAS by comparing characteristics of patients enrolled in a large multinational survey in relation to geographic origin, specialty of attending physician, detection of hemophagocytosis (HP), and outcome.

## Methods

Patient data were collected retrospectively by pediatric rheumatologists (PR) or pediatric hemato-oncologists (PHO). Clinical features, treatments and outcome were compared between groups by Mann-Whitney or chi-square tests. “Severe course” was defined as ICU admission or death.

## Results

362 patients with MAS in sJIA were collected by 95 investigators from 33 countries. 179 patients (49.4%) were enrolled in Europe (EU), 72 (19.9%) in North America (NA) and 111 (30.7%) in other continents (OC). 79 (21.8%) patients were included by PHO. HP was detected in 44% of patients and was not detected or looked for in 56%. Severe course was reported in 92 (25%) patients. Comparison by geographic origin showed a lower frequency of CNS disease in EU patients. NA physicians used more frequently ivIg and biologics. Patients entered by PHO had greater frequency of multiorgan failure and were given more commonly biologics and etoposide, whereas PR used more frequently cyclosporine (CsA). Patients with HP had shorter duration of sJIA at MAS onset, higher prevalence of hepatosplenomegaly, lower levels of platelets and fibrinogen and received more frequently CsA, ivIg and etoposide. Patients with severe course were older, had longer duration of sJIA at MAS onset, greater frequency of haemorrhages and CNS dysfunction, lower levels of ESR, albumin and fibrinogen, higher levels of LDH and D-dimer and were treated more commonly with CsA, ivIg and etoposide.

## Conclusion

Clinical and histopathologic features of MAS in sJIA were overall comparable among patients from different continents, whereas there was disparity in therapeutic choices made by specialists practicing in different geographic areas or fields. Patients with detection of HP or severe course had more acute clinical picture and were treated more aggressively.

## Disclosure of interest

None declared.

